# Case Report: MIS-C Temporarily Associated With COVID-19 Complicated by Reye's Syndrome

**DOI:** 10.3389/fped.2021.650697

**Published:** 2021-04-16

**Authors:** Fabrício Silva Pessoa, Eliza Maria da Costa Brito Lacerda, Valdênia Costa Gonçalves, Barbara Neiva Tanaka

**Affiliations:** ^1^Department of Microbial Biology, Ceuma University, São Luis, Brazil; ^2^Intermediate Care Unit, Dr. Odorico de Amaral Matos Children's Hospital, São Luis, Brazil; ^3^Pediatric Intensive Care Unit, Dr. Carlos Macieira High Complexity Hospital, São Luis, Brazil; ^4^Pediatric Infectious Diseases Department, Federal University of Maranhão Hospital (UFMA) University Hospital, São Luis, Brazil

**Keywords:** COVID-19, multisystem inflammatory syndrome in children, Reye syndrome, COVID-19 pandemic, SARS-CoV-2 (COVID-19)

## Abstract

We describe a 7-year-old child with multisystemic inflammatory syndrome that was temporarily associated with the novel coronavirus disease which evolved into serious illness, with coronary aneurysm, using human immunoglobulin and acetylsalicylic acid, in which clinical manifestations including hepatitis, convulsions, and coma were aggravated with Reye's syndrome. To date, there has been no report of the association of multisystemic inflammatory syndrome that is temporarily associated with the novel coronavirus disease and Reye's syndrome.

## Introduction

Multisystemic inflammatory syndrome in children (MIS-C) that is temporarily associated with the novel coronavirus disease 2019 (COVID-19) has been recently described as an inflammatory complication after exposure to the severe acute respiratory syndrome coronavirus 2 (SARS-CoV-2), with the possibility of serious and lethal complications, including coronary aneurysm (1). Reye's syndrome is a rare complication due to the use of aspirin, with onset following recovery from a viral disease (2).

This article describes the complete clinical course and follow-up data of a 7-year-old child treated at a pediatric intensive care unit (PICU) with severe MIS-C with coronary aneurysm, who was treated with human intravenous immunoglobulin (IVIG) and acetylsalicylic acid (ASA). The patient subsequently developed Reye's syndrome with life-threatening clinical presentations, including hepatitis, seizures, and coma. To our knowledge, there has been no reported association between MIS-C and Reye's syndrome.

## Case Report

**Figure 1:**
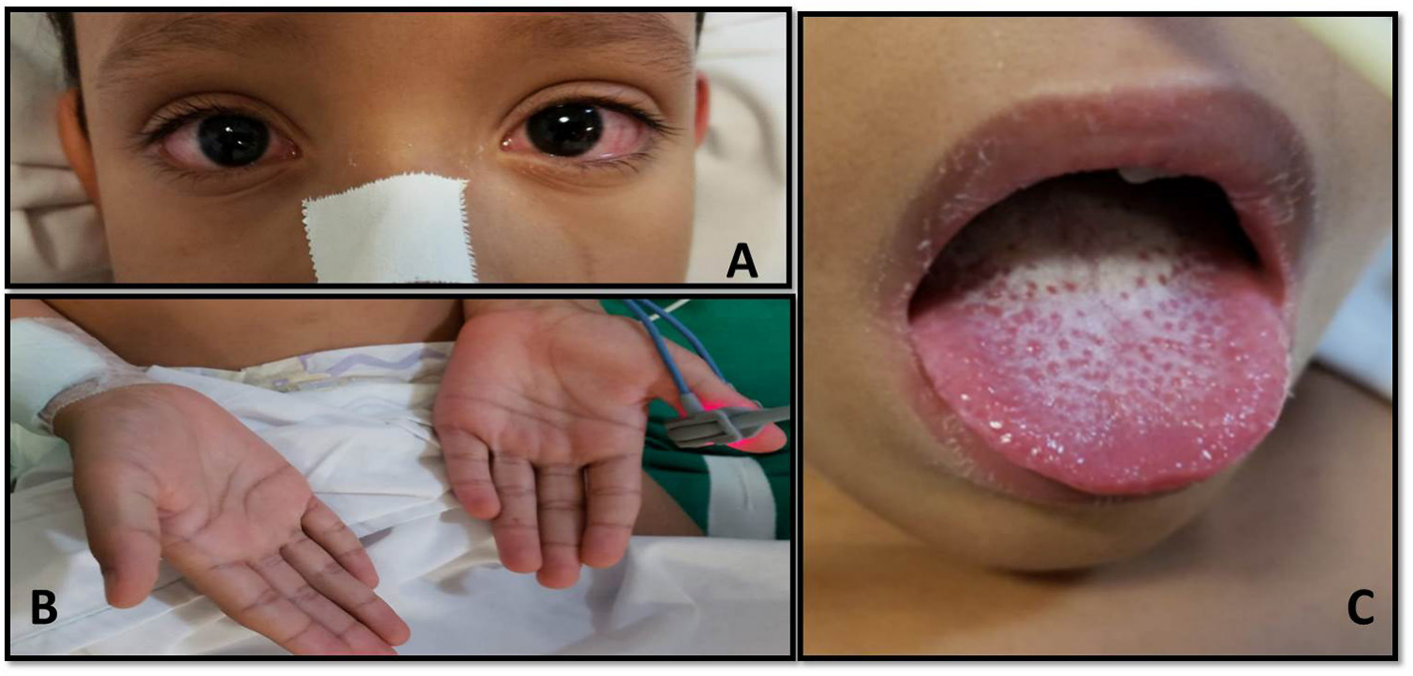
**(A)** Presence of bilateral, nonexudative conjunctival hyperemia. **(B)** Maculopapular lesions in the palmoplantar region. **(C)** Presence of mucosal changes with peeling lip and raspberry tongue.

A 7-year-old boy from the countryside of the state of Maranhão, northeastern Brazil, was admitted to the PICU having had fever for 6 days, headache, vomiting, maculopapular rash, severe abdominal pain, conjunctival hyperemia, photophobia, raspberry tongue, hyperemia with lip desquamation, right cervical lymphadenomegaly, and edema. In addition, he experienced mild tachypnea without respiratory symptoms when breathing normal room air (Figure 1).

On admission, the patient underwent a fluorescence immunoassay for SARS-CoV-2 to test for the presence of reagent immunoglobulin G (IgG) antibodies, with titers >30.0 AU/mL (normal, <1.4 AU/mL) and antibodies nonreactive to immunoglobulin M (IgM), with a titer of 0.1 AU (normal, <1.1 AU). The mother reported that the child had flu-like symptoms 1 month before admission.

Laboratory tests indicated inflammatory responses (Table 1). His C-reactive protein measured 96 mg/dL, blood count showed lymphopenia, anemia, and thrombocytopenia; hypoalbuminemia (2.3 g/dL); hypertriglyceridemia (262 mg/dL); and increased levels of transaminases, ferritin (1,812 ng/mL), d-dimer (8,500 ng/mL), and troponin (0.26 ng/mL). The patient's blood type was O positive. Serology tests for dengue IgG and IgM and anti-HIV were nonreactive. Serology tests for toxoplasmosis, cytomegalovirus, Epstein–Barr virus, herpes simplex 1 and 2, and rubella were all negative. Blood culture for aerobic and anaerobic microbes using selective media showed no bacterial growth. Urine culture also revealed no bacterial growth.

**Table 1:** Clinical laboratory results during the current hospitalization.

**Hospitalization day**	**11/06**	**12/06**	**13/06**	**14/06**	**17/06**	**27/06**	**29/06**	**30/06**	**Range & Units**
Hemoglobin (g/dL)	9.6	7.6	11.7	11.9	12.0	12.6	12.2	11.5	11.5–15.5
Hematocrit (%)	30.3	23.5	35.6	35.9	36.5	37.6	36.1	34.2	35–45
WBC (× 109/L)	20.0	7.47	13.13	11.4	10.31	4.03	8.62	8.29	3.20–10.0
NEU (× 109/L)	18.0	5.7	11.3	9.12	6.59	1.49	4.12	3.72	3.0–7.0
LYM (× 109/L)	0.86	0.93	1.37	1.69	2.29	2.10	3.53	3.20	1.5–4.0
PLT (× 109/L)	74	64	155	246	530	239	173	162	150–450
C-reactive protein (mg/dL)	18.3	10.5	7.4	4.0	1.5	2.43	4.18	4.13	<1.0
ALT (U/L)	74.1	41.9	47	–	31	–	479.5	459.9	10–40
AST (U/L)	190	53.1	43	–	34	–	1.117	1.073	10–40
LDH (U/L)	325	–	–	–	230	–	–	–	100–190
BUN (mg/dL)	76	25.1	23.8	23.7	30.0	–	21.6	19.1	16–40
SCR (mg/dL)	0.9	0.3	0.28	0.26	0.38	–	0.47	0.47	0.6–1.1
D-Dimer (ng/mL)	8.500	–	–	4.780	1.331	–	30.970	–	<500
Creatine phosphokinase (U/L)	81	–			19	–	199	283	32–190
CK-MB (U/L)	–	–	–	–	–	–	–	1.83	<5
Troponin (ng/mL)	0.26	–	–	0.07	0.04	–	6.5	5.5	<0.04
Ferritin (ng/mL)	1.385	–	–	385.6	321.9	–	16.500	–	30–300
BNP (pg/mL)	1.812	–	–	–		–	42.4	–	0–70
Triglycerides (mg/dL)	262	–	–	–	159	–	158	176	<160
Albumin (g/dL)	2.3	3.6			3.7	–	3.8	3.5	3.5–5.5
Myglobin (ng/mL)	–	–	–	–	–	–	34	–	<110
Fibrinogen (mg/dL)	–	–	–	–	–	–	–	130.4	200–400
Ammonia (150 μmol/L)	–	–	–	–	–	–	150	–	10–80

**Figure 2:**
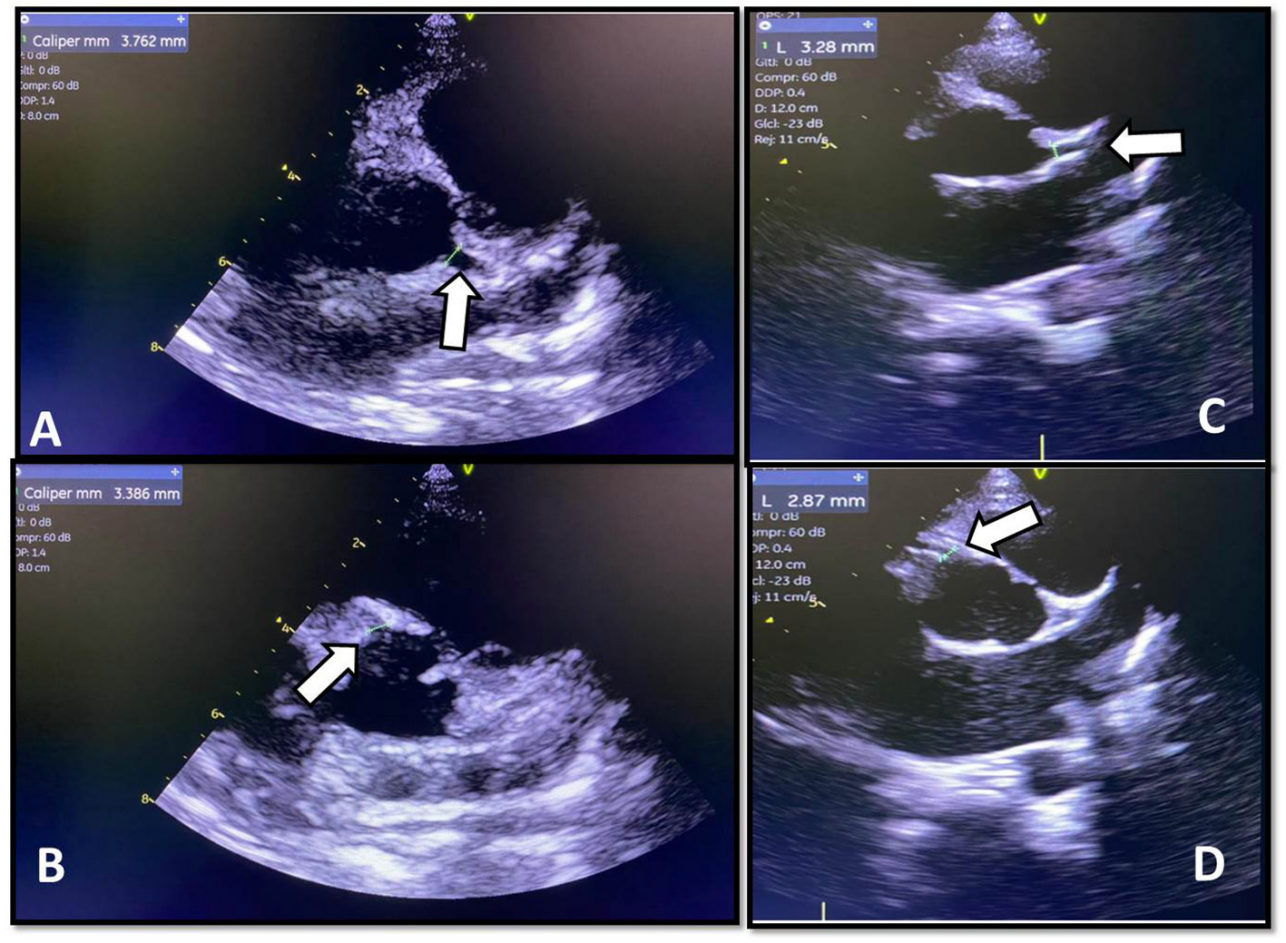
**(A)** Pretreatment echocardiographic images of the left main coronary artery measuring 3.762 mm (*Z* score +3.22). **(B)** Pretreatment echocardiographic images of the proximal right coronary measuring 3.38 mm (*Z* score +2.86). **(C)** Posttreatment echocardiographic images of the left main coronary artery measuring 3.28 mm (*Z* score +2.04). **(D)** Posttreatment echocardiographic images of the right proximal coronary artery measuring 2.87 mm (*Z* score +1.66).

Real-time polymerase chain reaction (PCR) of the nasopharyngeal swab for influenza A, influenza B, and respiratory syncytial virus revealed that these viruses as undetectable. PCR of nasopharyngeal and rectal samples showed SAR2-CoV-2 as undetectable. The patient had normal C3, C4, and CH50 serum complement levels. Other test results included negative direct Coombs, normal reticulocytes, and a normal simple urine test. The chest computed tomography (CT) scan showed moderate bilateral pleural effusion associated with fissure thickening on the right, interlobular septa thickening, mild infectious/inflammatory opacity, and mild pericardial effusion. The abdominal CT scan showed diffuse subcutaneous tissue edema and large amounts of free fluids in the abdominal and pelvic cavities. The transthoracic echocardiogram showed aneurysmal dilation of the right coronary and left main coronary arteries (Figure 2).

The patient received intensive support and was treated with antimicrobials (ceftriaxone, oxacillin, and azithromycin) and received intravenous human albumin. In addition, MIS-C was treated with a single IVIG dose of 2 g/kg by continuous infusion over 10 h, with the aim of promoting anti-inflammatory and immunomodulatory action and ASA, initially at the oral anti-inflammatory dose of 80 mg/kg per day (6/6 h) until the child became afebrile after 48 h and the oral maintenance dose was reduced to 5 mg/kg per day, single oral dose, as an antiplatelet agent, indicated by the presence of coronary dilations.

Two weeks after IVIG and ASA administration, while still hospitalized, the patient experienced a seizure crisis. His consciousness level lowered, and coma eventuated. He was intubated and managed with neuroprotective and anticonvulsant measures. The patient also had increased liver enzymes. Laboratory tests showed significant liver function damage (Table 1) and an ammonia level of 150 μmol/L. Cerebrospinal fluid (CSF) was collected with a cell count of 6/mm3, normal protein (33.9 mg/dL), and normal glucose (66.32 mg/dL). Other results included negative CSF Gram stain bacterioscopy and liquor culture without growth. Latex agglutination reactions were performed with specific antibodies for groups A, B, C, Y, and W-135 *Neisseria meningitidis, Streptococcus pneumoniae*, and type B *Haemophilus influenzae*, and these were all undetectable. Electroencephalogram showed no epileptiform activity. Blood was detected in the feces and fecal leukocytes. Skull CT demonstrated no abnormal changes.

Initially, the diagnosis of sepsis of hospital origin was considered, with meropenem and teicoplanin being prescribed. The patient received blood components, including red blood cells, fresh plasma, and vitamin K for severe coagulation disorders. In addition, he was administered sodium bicarbonate for metabolic acidosis, and ventilation control was provided. It was also necessary to administer vasoactive amines.

At that time, the patient was still using ASA; after clinical and laboratory investigation of the patient, the diagnosis of Reye's syndrome was given, as ASA is associated with such a syndrome, with the switch from ASA to clopidogrel, in one initial dose of 1 mg/kg per day, in order to maintain the antiplatelet activity.

The patient showed signs of improvement clinically. His liver enzymes decreased, and neurological condition improved. He was extubated and discharged from hospital after 2 weeks and followed up as an outpatient. A control echocardiogram was done and showed no coronary aneurysmal dilation (Figure 2).

## Discussion

This case report described a 7-year-old child with MIS-C, treated with IVIG and ASA, who subsequently developed Reye's syndrome with neurological and hepatic deteriorations, without other justified causes. The case presented a rare complication that was severe and potentially lethal, with critical progression and difficult management, but, ultimately, a satisfactory outcome.

Infections with SARS-CoV-2 in children are generally mild, but there may be complications associated with an inflammatory disorder, which can lead to serious illnesses and long-term side effects, referred to here as MIS-C. Most cases of MIS-C associated with COVID-19 are treated following the standard protocols for Kawasaki disease (3). Dufort et al. highlight that the incidence of MIS-C was 2 per 100,000 in people younger than 21 years (4).

MIS-C is a severe condition similar to Kawasaki disease, based on six main diagnostic elements: pediatric age, persistent fever, presence of laboratory markers of inflammation, signs or symptoms of organ dysfunctions, absence of an alternative diagnosis, and history of COVID-19 infection or exposure with possible cardiac and neurological presentations (5, 6). Several societies and organizations, such as the World Health Organization and the Centers for Disease Control and Prevention (CDC), have defined the diagnostic criteria of MIS-C (1). This case had a presentation that matched the diagnostic criteria of MIS-C, as the patient had fever for 6 days; headache; vomiting; maculopapular rash; severe abdominal pain; nonpurulent conjunctival hyperemia; photophobia; mucocutaneous inflammation signs (raspberry tongue, hyperemia with epithelial desquamation of the lips); presence of laboratory inflammatory markers, including erythrocyte sedimentation rate, C-reactive protein, D-dimer, and ferritin; right cervical lymphadenomegaly; and cardiac changes, as well as previous exposure to SARS-CoV-2 as confirmed by serology, and no other justified cause.

There are similarities between MIS-C and atypical Kawasaki disease; however, we observed some differences that can help to differentiate both situations. In this case, MIS-C generally presents in a higher incidence of gastrointestinal diseases, in children of older ages and with greater cardiac involvement (7). Despite the differential diagnoses between MIS-C and atypical Kawasaki disease being very close, the differences point to MIS-C, marked by the previous exposure to SARV-Cov-2, as a 7-year-old child, with gastrointestinal manifestations, represented by abdominal pain and, in addition, cardiac manifestations with coronary dilation, evidencing the importance of always performing this differential diagnosis in the management of these cases.

The presence of coronary aneurysm is a serious complication, well-described in Kawasaki disease, and present in MIC-S. Coronary aneurysm may progress to thrombosis, infarction, and cardiac arrhythmias that may persist for life and even lead to death. The timely treatment with IVIG until the 10th day of the disease reduced the possibilities of these complications (5, 8). The child described in this case report had MIC-S with cardiac changes as confirmed by echocardiography and was treated using IVIG and ASA.

Reye's syndrome begins within days of recovery from a viral disease wherein aspirin was administered. Reye's syndrome usually presents with an acute noninflammatory encephalopathy with fatty liver failure. It is a rare and potentially fatal pediatric disease. Affected children present with vomiting and mental confusion, rapid progression to coma, and death. Diagnosis of Reye's syndrome is based on clinical signs and laboratory tests (8).

Reye's syndrome is a known complication reported during the administration of ASA in children after a viral infection. Possible explanations for the pathophysiology of Reye's syndrome should be made. This syndrome is associated with a generalized disturbance in mitochondrial metabolism, resulting in metabolic failure in the liver and other tissues. Hypothetically, we can define that there are genetic factors, which can be modified by exogenous agents (in this case, ASA) and stimulated by a response to previous viral infection (9). In the case reported here, we describe a child who had a COVID-19 infection and subsequently progressed with MIC-S, presenting a cardiac complication (coronary aneurysm), with IVIG and ASA being administered and later progressing with liver failure and neurological condition, receiving the diagnosis of Reye's syndrome.

The CDC defined the diagnostic criteria for Reye's syndrome with three items: (1) acute noninflammatory encephalopathy (documented by changed consciousness); (2) CSF containing ≤ 8 leukocytes/mm (or a histological sample showing cerebral edema without perivascular or meningeal inflammation); and (3) liver disease (confirmed via liver biopsy/autopsy or a 3-fold or higher increase in serum glutamic-oxaloacetic transaminase, serum glutamic–pyruvic transaminase, and serum ammonia). In addition, Reye's syndrome is diagnosed when there are no other reasonable explanations for the brain and liver abnormalities (8).

This case report described a child with MIC-S, who underwent IVIG and ASA treatment and deteriorated after 1 week of treatment, with an altered level of consciousness, seizures, normal CSF, laboratory-proven hepatic changes, and no explanations to justify the brain and liver changes.

An important point that should be highlighted in the case was the prescription of ASA for the child because of the need to reduce coronary complications and thrombotic events. Ahmed et al. reported that up to 16.8% of all MIC-S cases reported the use of ASA (5).

Bianconi et al. described the use of ASA in COVID-19 and the possible complications from its anti-inflammatory and antithrombotic effects. The authors concluded that although the possibility of significant complications was rare, severe liver and brain injuries in children may result (i.e., Reye's syndrome) (10).

One of the important strategies used to ensure antithrombotic benefits and improve the clinical course of Reye's syndrome (given the need to maintain an antiplatelet agent and reduce the risk of complications) was to replace ASA with clopidogrel. Eleftheriou et al. reported that clopidogrel is an alternative antiplatelet agent that can be used in Kawasaki disease and therefore may be useful for MIC-S (11).

The literature highlights that the anatomopathological criterion for Reye's syndrome, with liver biopsy, may be useful, but the risk of complications, such as bleeding, is increased (12). In this reported case, hepatic changes were verified by tests that showed an increase in liver enzymes and ammonia.

The child underwent treatment with intensive support, initially for MIC-S and, subsequently, for Reye's syndrome. The patient progressed satisfactorily and demonstrated good clinical and laboratory results, reversed hepatic and neurological dysfunctions, and complete regression of the coronary aneurysm as confirmed during his outpatient follow-up.

Recent articles show that SARS-CoV-2 infection can complicate the onset of several autoimmune and autoinflammatory diseases, including MIS-C and Guillain-Barré syndrome, that need fundamental immunological therapies, such as immunoglobulin (13–16).

MIC-S is an inflammatory disease arising after exposure to SARS-CoV-2, may progress to systemic and vascular changes, and is potentially lethal. This description of a case of MIC-S, complicated by Reye's syndrome, resulted in a good clinical response after management and is unprecedented in the literature. Reye's syndrome should be considered in all pediatric cases of MIC-S treated with ASA.

## Data Availability Statement

The original contributions presented in the study are included in the article/supplementary material, further inquiries can be directed to the corresponding author/s.

## Ethics Statement

The studies involving human participants were reviewed and approved by the Research Ethics Committee of Universidade Ceuma (CEP) in compliance with the requirements of Resolution 466/2012 of the National Health Council that guides research involving human beings, directly or indirectly. The project was approved with CEP Opinion N°. 4,315,245. Written informed consent to participate in this study was provided by the participants' legal guardian/next of kin. Written informed consent was obtained from the minor(s)' legal guardian/next of kin for the publication of any potentially identifiable images or data included in this article.

## Author Contributions

FP, VG, BT, and EL conceptualized and designed the study, coordinated and supervised data collection, and drafted and critically reviewed the manuscript for important intellectual content. All authors approved the final manuscript as submitted and agree to be accountable for all aspects of the work.

## Conflict of Interest

The authors declare that the research was conducted in the absence of any commercial or financial relationships that could be construed as a potential conflict of interest.

## Abbreviations

MIS-C: Multisystemic inflammatory syndrome
COVID-19: Novel coronavirus disease
SARS-CoV-2: Severe acute respiratory syndrome coronavirus 2
PICU: Pediatric intensive care unit
IVIG: Human immunoglobulins
ASA: Acetylsalicylic acid.
